# Novel *PIK3CG* compound heterozygous variants cause inactivated PI3Kγ syndrome presenting as necrotizing enterocolitis in a preterm infant

**DOI:** 10.1016/j.gendis.2025.101618

**Published:** 2025-04-09

**Authors:** Wenting Zhang, Xiaoying Zhou, Bixia Zheng, Xinyi Yang, Yongcheng Ni, Dong Zhou, Chunli Wang

**Affiliations:** a Central Laboratory of Pediatrics, Affiliated Changzhou Children's Hospital of Nantong University, Changzhou, Jiangsu 213003, China; b Pharmaceutical Laboratory, Asthma and Bronchitis Research Center of Changzhou, Changzhou, Jiangsu 213003, China; c Department of Neonatology, Affiliated Changzhou Children's Hospital of Nantong University, Changzhou, Jiangsu 213003, China; d Nanjing Key Laboratory of Pediatrics, Children's Hospital of Nanjing Medical University, Nanjing, Jiangsu 210008, China; e Department of Pediatric Intensive Care, Affiliated Changzhou Children's Hospital of Nantong University, Changzhou, Jiangsu 213003, China

Inactivated phosphoinositide 3-kinase gamma (PI3Kγ) syndrome (IPGS; OMIM #619802), an autosomal recessive immunologic disorder first described by Takeda et al in 2019, classically manifests in childhood with recurrent infections, pneumonia, and colitis.^1^ This disorder is caused by biallelic loss-of-function variants in the *PIK3CG* (OMIM ∗601,232), located at 7q22.3, encoding the catalytic subunit p110γ of the PI3Kγ enzyme. The p110γ subunit, predominately expressed in immune cells and responsible for chemotaxis, reactive oxygen species (ROS) generation, and cytokine generation, also maintains critical roles in endothelial cells, neurons, cardiomyocytes, and lung cells.^2^^,^^3^ Pathogenic variants in *PIK3CG* disrupt PI3K signaling, leading to immune dysregulation characterized by antibody deficiency, excessive T cell infiltration in the lungs/intestines, and significantly disrupted levels of T regulatory cells.^1^^,^^4^

Here, we report a 30-week preterm neonate (birth weight 1500 g) exhibiting classic features of prematurity, including low birth weight and respiratory distress syndrome. Although the initially therapeutic response was favorable, the infant later developed severe pneumonia and necrotizing enterocolitis, ultimately succumbing to septic shock following infection. Respiratory distress occurred 3 h after birth and was successfully managed with non-invasive continuous positive airway pressure ventilatory support. Enteral feeding was gradually increased to 23 mL every 3 h, enabling antibiotics de-escalation. After 20 days of treatment, the infant developed abdominal distention and hematochezia. Laboratory investigations confirmed a *Klebsiella pneumoniae* infection, and abdominal X-rays confirmed the diagnosis of neonatal necrotizing enterocolitis, which proved refractory to conservative management. On day 23 of life, surgical intervention became necessary, involving partial ileal resection, double-barrel ileostomy creation, and abdominal drainage. However, within 24 h postoperatively, the exteriorized intestinal segment exhibited necrotic changes with tissue induration, necessitating emergency reoperation on postnatal day 24. A pre-operative chest computed tomography scan showed inflammation of the right upper lobe of the lung with atelectasis (Fig. 1A). The detailed histopathological findings from both surgical specimens are presented in Figure 1B. After the surgeries, the infant suffered from two recurrent infections, with laboratory tests showing elevated C-reactive protein, procalcitonin, white blood cells, and neutrophils, accompanied by a significant decrease in lymphocytes. Based on microbiological monitoring, the antimicrobial therapy was adjusted from a combination of meropenem and vancomycin to cefoperazone-sulbactam. This therapeutic approach gradually achieved clinical stabilization, enabling discharge on day 73 of life. Tragically, only 8 days after discharge, the infant developed a recurrent infection manifested by hemorrhagic discharge from the stoma and darkening of the exposed intestinal tube, ultimately necessitating readmission for symptom-targeted supportive care*.* The patient succumbed on postnatal day 81.

**Figure 1:**
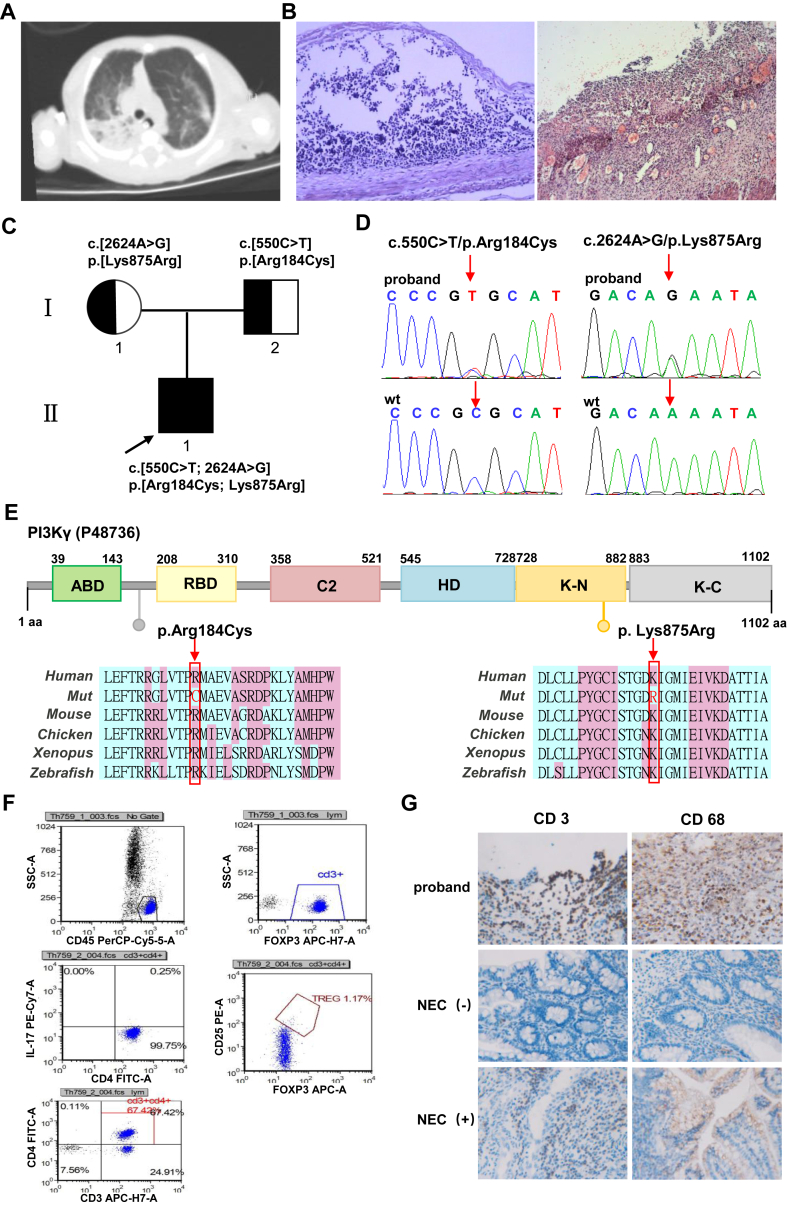
Clinical findings, genetic analysis, and functional investigations. **(A)** A chest computed tomography scan showed pulmonary inflammation in the patient. **(B)** Histomorphology analysis of ileum tissue resected during ilectomy (left) and ileostomy necrosis (right) showing necrotizing enterocolitis (NEC)-like damage (hematoxylin and eosin (HE) staining, 100 × ). Injured epithelium barrier integrity, congestion, and inflammatory cell infiltration were observed in the ileum of the patient. **(C)** Pedigree of the family with *PIK3CG* variants. The proband (II-1, solid symbol) is affected. The squares indicate male members (proband and father, I-2), and the circle represents the mother (I-1). **(D)** Sanger sequencing showed two missense variants (arrows): c.550C > T and c.2624A > G in the *PIK3CK* gene (top). The wild-type sequence is also shown (bottom). **(E)** The p. Arg184Cys and p. Lys875Arg variants are located in PI3Kγ corresponding domains (top). Multiple-sequence alignment demonstrates high evolutionary conservation of the affected amino acids from humans to zebrafish (bottom). **(F)** Flowcytometry analysis showed the reduced percentage of T regulatory cells (Tregs) within the CD4^+^ T cell population (reference range: 2.17%–7.94%). **(G)** Immunohistochemistry (IHC, 400 × ) analysis demonstrated elevated densities of CD3^+^ and CD68^+^ cells in the proband's ileum tissue compared with specimens from other infants with NEC or intestinal atresia (NEC (−)).

Due to the patient's clinical history, genomic DNA was extracted and subjected to whole exome sequencing. Two novel missense variants (NM_001282426, c.550C > T (p.Arg184Cys); c.2624A > G (p.Lys875Arg)) in the *PIK3CG* gene were identified in the proband. Segregation analysis by Sanger sequencing confirmed the paternal origin of the c.550C > T (p.Arg184Cys) variant (individual I-2) and maternal transmission of the c.2624A > G (p.Lys875Arg) variant (individual I-1) (Fig. 1C, D). Furthermore, these missense variants showed high conservation of each amino acid altered from humans to zebrafish (Fig. 1E) and were absent in the 1000 Genomes, ExAC, and gnomAD databases. According to the American College of Medical Genetics and Genomics (ACMG) guidelines, both c.550C > T (p.Arg184Cys) (PM2_P + PP3) and c.2624A > G (p.Lys875Arg) (PM2_P) were classified as variants of uncertain significance.

Based on the genetic testing analysis, inactivated PI3Kγ syndrome was suspected. To validate this hypothesis, we conducted targeted examinations based on the patient's clinical manifestations. Firstly, after infection, the infant's blood routine examinations consistently showed a significant decrease in lymphocyte percentage (from 20.1% on day 27 of life to 60% on day 61 of life, with a minimum of 3.8%). Further analysis of lymphocyte subpopulations demonstrated a decreased percentage of T regulatory cells within CD4^+^ T cell population (Fig. 1F). Finally, immunohistochemical staining of the patient's postoperative intestinal tissue for macrophages (CD68^+^) and lymphocytes (CD3^+^) revealed significantly increased infiltration of both cell types in the necrotic intestinal sections compared with tissues from other patients with necrotizing enterocolitis and controls with intestinal atresia (Fig. 1G). The collective evidence from genetic testing, clinical findings, and bioinformatic analysis strongly supported the diagnosis of *PIK3CG* variants associated with PI3Kγ deficiency syndrome.

This study reports a case of inactivated PI3Kγ syndrome with the earliest onset of symptoms. The patient's death due to severe infection emphasizes the critical importance of early diagnosis and intervention. Notably, this is the first reported case in China with compound heterozygous *PI3KCG* variants (c.550C > T (p.Arg184Cys); c.2624A > G (p.Lys875Arg)), expanding this gene's pathogenic variant spectrum. However, the pathogenic mechanisms underlying these two missense variants require further experimental validation.

## CRediT authorship contribution statement

**Wenting Zhang:** Writing – original draft, Funding acquisition, Conceptualization. **Xiaoying Zhou:** Writing – original draft, Conceptualization. **Bixia Zheng:** Formal analysis. **Xinyi Yang:** Data curation. **Yongcheng Ni:** Data curation. **Dong Zhou:** Data curation. **Chunli Wang:** Writing – review & editing, Formal analysis, Conceptualization.

## Ethics declaration

This research protocol has been reviewed and approved by the Ethics Committee of Affiliated Changzhou Children's Hospital of Nantong University (Changzhou, China) (ethics approval number: 2023–002). Written informed consents were obtained from the patients/participants in this study.

## Funding

This study was supported by the 10.13039/501100002858China Postdoctoral Science Foundation (No. 2021M700546), Changzhou Sci&Tech Program (China) (No. CE20235066, CE20225052), Key project of Changzhou Medical Center Affiliated to Nanjing Medical University (China) (No. CMCM202314), and Clinical Research Project of Nantong University (China) (No. 2019LY029).

## Conflict of interests

The authors declared no conflict of interests.
