# Supplementary material for: Novel PIK3CG compound heterozygous variants cause inactivated PI3Kγ syndrome presenting as necrotizing enterocolitis in a preterm infant
Source: Genes Dis. 2025 Apr 9;13(2):101618. doi: 10.1016/j.gendis.2025.101618 (PMC12596569; doi:10.1016/j.gendis.2025.101618)
Supplement: Multimedia component 1 [file mmc1.docx]

Detailed materials & methods

**Genetic analysis and bioinformatics predictions**

Peripheral blood samples from the pediatric patient and their family members were collected for Whole Exome Sequencing (WES). The WES procedure was carried out as previously described^[5]^. In brief, genomicDNA was isolated from peripheral blood by a DNA isolation kit (Tiangen, China) and was sequenced using WES by Illumina sequencers (HiSeq 2500 sequencing platform). Using BWA software, the sequences were aligned to the reference human genome (hg19 build). Single nucleotide variation (SNV) and insertions/deletions (INDELs) were filtered by GATK software (https://software.broadinstitute.org/gatk/) and were annotated by ANNOVAR software. The variants with allele frequencies greater than 1% in the public databases, including Genome Aggregation Database (gnomAD) datasets, dbSNP databases, 1000 Genomes MAF and ExAC were excluded. Pathogenicity analysis of the variants is conducted using Mutation Taster (http://www.mutationtaster.org/), PolyPhen2 (http://genetics.bwh.harvard.edu/pph2/), Revel score (http://www.genome.ucsc.edu/cgi-bin/hgTrackUi?db=hg19&g=revel), and SIFT (http://sift.bii.a-star.edu.sg/). The MEME Suite motif analysis (http://meme-suite.org/tools/meme) was used to identify the motifs. The candidate variants were clarified accordancing to the American College of Medical Genetics and Genomics (ACMG) guidelines and then validated by Sanger sequencing.

This research protocol has been approved by the Ethics Committee of Affiliated Changzhou Children's Hospital of Nantong University. All variations are based on the NCBI reference sequence of *PIK3CG* (NM_001282426).

**Histomorphological and immunohistochemical staining**

The surgically resected intestinal segments obtained from the patient were embedded in paraffin blocks . The pathological examination was performed by haematoxylin and eosin (H&E) staining. The infiltration of T cells (CD3^+^) and macrophages (CD68^+^) were determined by immunohistochemical (IHC) staining using BenchMark GX automated stainer (Roche). Rat monoclonal anti-CD3 (1:200, Abcam) and rabbit monoclonal anti-CD68 antibodies (1:8000, Abcam) were used as primary antibodies. Horseradish peroxidase-labelled secondary antibodies were used accordingly, and the diaminobenzidine (DAB) detection kit (Ventana Medical Systems, Inc., Tucson, AZ, USA) was used for visualization. The photomicrographs were taken with CX33 upright microscope (Olympus, USA) .

### Flowcytometry

Treg and Th17 population were determined using flowcytometry. Peripheral blood samples 4 mL were obtained from the patient. Peripheral blood mononuclear cells (PBMCs) were obtained using Ficoll density gradient. After centrifugation and discard of supernant, the PBMCs were suspended at the density of 1×10^7^ cells/mL in completed culture medium which including RPMI 1640 medium, fetal bovine serum and cell activation cocktail (with brefeldin A) (9: 1: 0.02, vol/vol). 1mL of PBMCs suspension was added to a 24-well plate and incubated for 6h (5%CO_2_, 37℃). After the incubation and centrifugation, the cells were then incubated with PerCP-conjugated anti-CD45 (5 µL), APC-H7-conjugated anti-CD3 (5 µL) and FITC-conjugated anti-CD4 (5 µL) monoclonal antibodies (mAbs) at room temperature (RT) for 20 min. After the surface staining, the cell culture was centrifuged, and the supernatant was discarded. After fixation/permeabilization according to the manufacturer’s instructions, the cells were incubated with APC-conjugated anti-Foxp3 (5 µL) and PE-Cy7-conjugated anti-IL-17 (5 µL) in the dark at 4℃ for 30 min. All the Abs were from BD Biosciences (USA). Isotype-matched negative controls were used to enable compensation correction. After washing with PBS, the marked cells were analyzed using FACS Calibur (BD Biosciences, USA).
